# Regularized Embedded Multiple Kernel Dimensionality Reduction for Mine Signal Processing

**DOI:** 10.1155/2016/4920670

**Published:** 2016-05-09

**Authors:** Shuang Li, Bing Liu, Chen Zhang

**Affiliations:** ^1^School of Management, China University of Mining and Technology, Xuzhou, Jiangsu 221116, China; ^2^School of Computer Science and Technology, China University of Mining and Technology, Xuzhou, Jiangsu 221116, China

## Abstract

Traditional multiple kernel dimensionality reduction models are generally based on graph embedding and manifold assumption. But such assumption might be invalid for some high-dimensional or sparse data due to the curse of dimensionality, which has a negative influence on the performance of multiple kernel learning. In addition, some models might be ill-posed if the rank of matrices in their objective functions was not high enough. To address these issues, we extend the traditional graph embedding framework and propose a novel regularized embedded multiple kernel dimensionality reduction method. Different from the conventional convex relaxation technique, the proposed algorithm directly takes advantage of a binary search and an alternative optimization scheme to obtain optimal solutions efficiently. The experimental results demonstrate the effectiveness of the proposed method for supervised, unsupervised, and semisupervised scenarios.

## 1. Introduction

Dimensionality reduction (DR) methods in supervised, unsupervised, and semisupervised learning tasks have attracted much attention in computer vision and pattern recognition [1–6]. These methods are often considered as feature extraction methods for high-dimensional signals from various application fields, such as transportation, communications, plants, and mines. Unsupervised dimensionality reduction, such as principle component analysis (PCA) [7], does not utilize any label information. Linear discriminant analysis (LDA) is a popular supervised dimensionality reduction method, which derives a projection from simultaneously maximizing the between-class scatter and minimizing the within-class scatter. Semisupervised dimensionality reduction, such as semisupervised discriminant analysis (SDA) [8], makes good use of labeled data while preserving the intrinsic geometric structures of unlabeled data.

In order to handle the data sampled from a low-dimensional manifold, some nonlinear dimensionality reduction methods, such as isometric feature mapping (ISOMAP) [9], locally linear embedding (LLE) [10], and Laplacian Eigenmap (LE) [11], introduce manifold assumption into dimensionality reduction and aim to maximally preserve certain interpoint relationships. But these methods cannot address the out-of-sample extension problem. Thus, locality preserving projections (LPP), as a linear approximation of LE [12], were proposed to both uncover the data manifold and provide out-of-sample extensions. These dimensionality reduction methods could be unified under a framework called graph embedding [13]. To achieve significant improvements, it is feasible to kernelize a certain type of linear methods into nonlinear ones [14–18]. But, the performances of the kernelized versions heavily rely on the selections of kernel functions. With inappropriate kernels, the performances will be degraded and become even worse.

Recently, the advantage of using multiple kernels instead of only one kernel for dimensionality reduction has been demonstrated [15, 19]. Multiple kernel learning for dimensionality reduction (MKL-DR) was proposed to learn an appropriate kernel from the multiple base kernels and a transformation into a lower dimensionality space simultaneously [20]. But, MKL-DR relaxes a nonconvex quadratically constrained quadratic programming (QCQP) into a semidefinite programming (SDP), which is very time-consuming and has a negative effect on its performance. Recently, a multiple kernel learning method called MKL-TR was proposed to improve the performance of MKL-DR [21]. MKL-TR formulates multiple kernel learning for dimensionality reduction as a trace ratio maximization problem. But both MKL-DR and MKL-TR need to iteratively compute generalized eigendecomposition of dense matrices. Motivated by the efficiency of spectral regression, a fast multiple kernel dimensionality reduction method, termed as MKL-SRTR, was presented to avoid generalized eigendecomposition of dense matrices [22]. It is more efficient than MKL-DR and MKL-TR by virtue of spectral regression. Since MKL-DR, MKL-TR, and MKL-SRTR are all based on graph embedding and manifold assumption, they cannot cope with manifold assumption invalidation. In addition, MKL-DR and MKL-SRTR might be ill-posed if the rank of matrices in their objective functions was not high enough [21].

Since spectral clustering and multiple kernel dimensionality reduction have the same form of optimization based on the manifold assumption, motivated by the spectral embedded clustering framework proposed in [22], we firstly extend the traditional graph embedding framework by incorporating linear regularization terms into its model, termed as extended graph embedding (EGE). Secondly, we introduce multiple kernel learning into EGE (termed as MKL-EGE) to improve the performance of single kernel DR. Compared with traditional multiple kernel dimensionality reduction methods, such as MKL-SRTR, the proposed method not only solves the ill-posed problems but also is more robust against high-dimensional or sparse data. Furthermore, our method directly utilizes a binary search and an alternative optimization scheme to obtain optimal solutions. The experimental results demonstrate that the proposed method achieves better or similar performance compared to other algorithms for supervised, unsupervised, and semisupervised settings.

The remainder of the paper is structured as follows. In Section 2, we briefly introduce the related work. We provide the MKL-EGE framework and the optimization process in Section 3. The experimental results are shown in Section 4. Finally, we give the related conclusions in Section 5. In order to avoid confusion, we give a list of the main notations used in this paper in Notations.

## 2. Graph Embedding and Its Extension

### 2.1. Graph Embedding

Specifically, denote an undirected weighted graph by **G** = {**X**, **W**}, where **X** = [**x**_1_, **x**_2_,…,**x**_*n*_]^*T*^ ∈ *ℝ* ^*n*×*d*^ is a vertex set and **W** ∈ *ℝ* ^*n*×*n*^ represents an affinity matrix. Each entry *W*_*ij*_ of the symmetric matrix **W** is the edge weight that characterizes the similarity between a pair of vertices of **G**. A dimensionality reduction scheme aims at finding a low-dimensional subspace **F** = [**f**_1_, **f**_2_,…,**f**_*n*_]^*T*^ (**f**_*i*_ ∈ *ℝ* ^*c*^, *c* ≪ *d*) by a complete graph **G** whose vertices are over **X**. The purpose of graph embedding is to represent each vertex of a graph as a low-dimensional vector and preserves similarities between the vertex pairs. The optimal **F** could be obtained by solving

(1) min⁡F tr⁡FTLFs.t. tr⁡FTDF=1  or  tr⁡FTL′F=1,

where **L** = **D** − **W** is the graph Laplacian matrix of **G** and **D** = diag(**D**_1_,…, **D**_*n*_) is a diagonal matrix with the diagonal elements defined as **D**_*i*_ = ∑_*j*=1_^*n*^ **w**_*ij*_. **L**′ = **D**′ − **W**′ is the graph Laplacian matrix of another weighted graph **G**′.

By specifying **W** and **D** (or **W** and **W**′), the PCA, ISOMAP, LLE, LPP, LDA, local discriminant embedding (LDE), marginal Fisher analysis (MFA) [13], and spectral regression (SR) [23, 24] can all be expressed as graph embedding. Since **L** = **D** − **W** and the constraint tr⁡(**F** ^*T*^ **D** **F**) = 1 are commonly used, in this paper, we mainly discuss the following form of graph embedding:

(2) max⁡F tr⁡FTWFs.t. tr⁡FTDF=1

which usually relaxes to the following objective function:

(3) max⁡FTF=Ic tr⁡FTWFtr⁡FTDF.

### 2.2. Extended Graph Embedding

The term tr⁡(**F** ^*T*^ **L** **F**) in problem (4) is actually derived based on the manifold assumption [25]. However, for high-dimensional or sparse data, this assumption may not hold due to the bias caused by the curse of dimensionality. Thus, the low-dimensional manifold structure cannot be exploited by the inaccurate similarity matrix, which would result in the performance degradation of graph embedding.

To address this issue, we try to improve traditional graph embedding framework. Notice that the term tr⁡(**F** ^*T*^ **L** **F**) can be regarded as the objective function of spectral clustering; we use the spectral embedded clustering method proposed in [26] to extend the graph embedding framework. Specifically, we minimize the following objective function:

(4) min⁡F,W,b,FTF=Ic tr⁡FTLF+μXTW+1nbT−F2+γgtrWTWtr⁡FTDF,

where *μ* and *γ*_*g*_ are two regularization parameters, 1_*n*_ denotes the *n* × 1 vectors of all 1s, and the second term characterizes the mismatch between the low-dimensional feature matrix **F** and the low-dimensional representation of the data.

**Theorem 1 .**

The optimization problem (4) can be transformed into the following minimization problem:

(5) minFTF=Ic tr⁡FTL~Ftr⁡FTDF,

where $\tilde{L}=L+\mu L_{s}$ and **L**_*s*_ = **I**_*n*_ − (1/*n*)1_*n*_1_*n*_^*T*^ − **X**(**X** ^*T*^ **X** + *γ*_*g*_ **I**_*d*_)^−1^ **X** ^*T*^. **I**_*n*_ and **I**_*d*_ represent the identity matrix of size n by n and size d by d, respectively.

**Proof**

By setting the derivatives of the objective function (4) with respect to **W** and **b** to zeros, we have

(6) b=1nFT1n,W=XTX+γgId−1XTF.

By substituting **W** and **b** in (4) by (6), the optimization problem (4) becomes

(7) minFTF=Ic tr⁡FTL+μLsFtr⁡FTDF,

where **L**_*s*_ = **I**_*n*_ − (1/*n*)1_*n*_1_*n*_^*T*^ − **X**(**X** ^*T*^ **X** + *γ*_*g*_ **I**_*d*_)^−1^ **X** ^*T*^. This completes the proof of Theorem 1.

From problem (5), we can find that the form of EGE is similar to that of GE and GE is a special case of EGE when *μ* = 0. $\tilde{L}=L+\mu L_{s}$ can be regarded as a correction of the graph Laplacian matrix **L** for high-dimensional data.

Since **L** = **D** − **W**, problem (5) can be transformed into the following form:

(8) maxFTF=Ic tr⁡FTW−μLsFtr⁡FTDF.

## 3. Multiple Kernel Learning Based on EGE and Trace Ratio Maximization

Since MKL-DR, MKL-TR, and MKL-SRTR can be viewed as multiple kernel versions of graph embedding, it is natural to establish a multiple kernel learning framework for dimensionality reduction based on EGE.

### 3.1. Formulation

Suppose the ensemble kernel *𝕂* is generated by linearly combining the base kernels {**K**_*m*_}_*m*=1_^*M*^; that is, *𝕂* = ∑_*m*=1_^*M*^ *β*_*m*_ **K**_*m*_, where *β*_*m*_ ≥ 0 and **K**_*m*_ = {*k*_*m*_(**x**_*i*_, **x**_*j*_)}_*i*,*j*=1_^*n*^. We can find a sample coefficient matrix **A** and a kernel weight vector **β** by the following trace ratio optimization problem based on extended graph embedding:

(9) max⁡A,β tr⁡ATKW−μLsKAtr⁡ATKDKAs.t. ATA=I βm≥0,m=1,2,…,M, ∑mMβm=1,

where

(10) A=α1α2⋯αc∈Rn×c,α=α1,…,αnT∈Rn,β=β1,…,βMT∈RM,Ki=k11,i⋯kM1,i⋮⋱⋮k1n,i⋯kMn,i∈Rn×M.

It should be noted that dimensionality reduction based trace ratio optimization tends to overfitting [27, 28]. To address this issue, a regularization term tr⁡(**A** ^*T*^ *λ* **I** **A**) is added to the denominator of problem (9) to ensure that *𝕂* **D** *𝕂* + *λ* **I** is of full rank. Hence, the objective function could be expressed as follows:

(11) max⁡A,β tr⁡ATKW−μLsKAtr⁡ATKDK+λIA

(12) s.t. ATA=I

(13)  βm≥0,m=1,2,…,M,

(14)  ∑mMβm=1.

Compared with MKL-SRTR, the proposed method is based on the extended graph embedding framework. Thus, it has more robustness against high-dimensional or sparse data. In addition, our method avoids ill-posed problems.

### 3.2. Method

To optimize our objective function, the following function that satisfies constraints (13)–(15) is defined:

(15) fr=max⁡A,β sr,A,β=max⁡A,β tr⁡ATKW−μLsK−rKDK−rλIA=max⁡A,β tr⁡ATKW−μLs−rDK−rλIA.

The optimal value of the objective function in (15) is the root of the function *f*(*r*) = max⁡_**A**^*T*^**A**=**I**_⁡tr⁡(**A** ^*T*^(*𝕂*(**W** − *μ* **L**_*s*_ − *r* **D**)*𝕂* − *rλ* **I**)**A**) [27, 28]. Based on (15), we update *r*, **A**, and **β** alternately.

*On Optimizing* **A** *and r*. By fixing **β**, optimization problem (11) is simplified to

(16) max⁡ATA=I tr⁡ATS1Atr⁡ATS2A,

where

(17) S1=KW−μLsK,

(18) S2=KDK+λI.

Thus, a binary search (giving a lower bound and an upper bound) is used to seek *r* ^*∗*^ such that *f*(*r* ^*∗*^) = 0. The value of *f*(*r*) can be easily calculated as the sum of the first *c* largest eigenvalues of **S**_1_ − *r* **S**_2_.  Optimal **A** ^*∗*^ is finally obtained by performing the eigenvalue decomposition of **S**_1_ − *r* ^*∗*^ **S**_2_.

*On Optimizing* **β**. By fixing **A** and *r*, **β** can be obtained by solving the following optimization problem:

(19) max⁡β tr⁡ATKW−μLs−rDKA,s.t. βm≥0,m=1,2,…,M, ∑mMβm=1.

We define a function with given **A** and *r* as follows:

(20) $$\begin{array}{c} Q\left(\;\beta \;\right)=\mathrm{tr}\left(\;A^{T}K\left(\;W-\mu L_{s}-rD\;\right)KA\;\right) \end{array}$$

and we have

(21) $$\begin{array}{c} \frac{\partial Q}{\partial \beta_{m}}=\mathrm{tr}\left(\;{2A}^{T}K_{m}\left(\;W-\mu L_{s}-rD\;\right)KA\;\right). \end{array}$$

Thus, **β** can be determined by updating the projections of **β** in the direction of ∂**Q**/∂**β**. Finally, we define a quadratic programming to satisfy the constraint ∑_*m*_^*M*^ *β*_*m*_ = 1 as

(22) $$\begin{array}{c} G\left(\;\beta \;\right)=\underset{h^{T}1=1,h\geq 0}{\arg\min}{\left\|\;h-\beta \;\right\|}_{2}^{2}, \end{array}$$

where 1 denotes *n* × 1 unit vector.

### 3.3. Algorithms

The proposed algorithm based on EGE and regularized trace ratio, termed as MKL-EGE, is described in Algorithm 1. As can be seen from Algorithm 1, MKL-EGE utilizes a binary search in inner iterations to speed up convergence and adopts updating **A** and **β** alternately in outer iterations to seek optimal solutions. Since the proposed algorithm cannot guarantee obtaining the optimal solution *r* ^*∗*^ exactly, we terminate it within a maximum iteration and choose the best result.

### 3.4. Computational Complexity

For MKL-EGE, the computational complexity of inner iterations is *O*(iter_2_ *n* ^3^), where iter_2_ is maximum number of inner iterations. Thus, the computational complexity of the whole algorithm is *O*(iter_1_(iter_2_ *n* ^3^ + *n* ^3^)), where iter_1_ is maximum number of outer iterations. MKL-DR needs to solve the SDP problem in each iteration, which is as high as *O*(*n* ^6.5^) [20].

The computational complexity of MKL-TR decreases to *O*(iter_1_(iter_2_(*cn* ^2^ + *n* ^3^) + *n* ^3^)) [21]. Since MKL-EGE only needs a small number of iterations to converge, the computational complexity of our method is much lower than that of MKL-DR and MKL-TR.

### 3.5. Unseen Sample Embedding

After accomplishing the training procedure of MKL-EGE, we can project a new sample **v** into the learned subspace by

(23) v⟶ATKvβ,where  Kv∈Rn×M,  Kvi,m=kmxi,v.

## 4. Experiments

We compared the proposed MKL-EGE algorithm with MKL-DR [20], MKL-TR [21], and MKL-SRTR [22] on UCI datasets (Sonar, Ionosphere, and Isolet), face recognition datasets (Yale, PIE, and ORL), digits recognition datasets (USPS and MNIST), object recognition datasets (COIL-20), and text datasets (20 newsgroups). We randomly selected 300 samples from each digit for the USPS dataset and used digits 3, 6, and 8 for the MNIST dataset. For 20 newsgroups datasets, four largest topics (comp, rec, sci, and talk) were selected as high-dimensional datasets. For all datasets, we randomly selected samples to form training and testing sets with ratio 1 : 1. The basic information of datasets is listed in Table 1. All the experiments were performed in MATLAB R2013a running in a 3.10 GHZ Intel Core*™* i5-2400 with 4-GB RAM.

For all datasets, we first normalized the values of the data vector to the range [0,1] and used 10 RBF base kernels, whose *σ* values are set as 0.10, 0.20, 0.40, 0.80, 1.60, 3.20, 6.40, 12.80, 25.60, and 51.20, respectively. In all experiments, we set* t*_1 = 0.5 and *ε* = 0.001. The parameter* k* of* k*-nearest-neighbor graph is set to 5 empirically. For fair comparisons, we set the parameters *λ* as 0.5 for MKL-TR and MKL-EGE. For MKL-SRTR and MKL-EGE, we conducted a search of the optimal parameters *γ*, *γ*_*g*_, and *μ* on {10^−9^, 10^−6^, 10^−3^,…,10^0^,…,10^3^, 10^6^, 10^9^} by using fivefold cross-validation and reported the best experimental results.

### 4.1. Experiments on Supervised Learning

The maximum number of iterations for all algorithms is set as 20. For MKL-DR, MKL-SRTR, and MKL-EGE, the affinity matrix **W** = [*w*_*ij*_] is defined as

(24) $$\begin{array}{c} w_{ij}=\left\{\begin{array}{cc} \frac{1}{n_{y_{i}}}, & \text{if }y_{i}=y_{j},\\ 0, & otherwise. \end{array}\right. \end{array}$$

For MKL-TR, we set **M** = **Η**(**H** ^*T*^ **Η**)^†^ **H** ^*T*^ − (1/**n**)11^*T*^ and **N** = **I** − **Η**(**H** ^*T*^ **Η**)^†^ **H** ^*T*^, where **Η** represents the indicator matrix with **Η**_*ij*_ = 1 if **x**_*i*_ belongs to class *j* and 0 otherwise. For MKL-DR, the elements of another affinity matrix **W**′ are all set as 1/**N**. The final reduced dimension is *c* − 1 for all algorithms. We used libSVM [29] with linear kernel to classify the embedding data. All experiments were independently carried out over 20 times.

The mean classification accuracies and the standard deviations of different algorithms are displayed in Table 2. As can be seen from Table 2, MKL-EGE significantly outperforms MKL-DR, MKL-TR, and MKL-SRTR in most datasets, which achieves 11 best recognition rates among all 13 datasets. In particular, the performance of MKL-EGE is much better than that of other algorithms on high-dimensional datasets such as Yale, PIE, ORL, and COIL-20. This is due to the fact that MKL-EGE incorporates EGE and linear regularization terms into its model, which is effective for handling high-dimensional data and can avoid overfitting. Consequently, MKL-EGE is more robust than other algorithms based on traditional graph embedding. In addition, the performance of MKL-EGE is very close to that of MKL-TR and MKL-SRTR on low-dimensional dataset, such as Ionosphere, which shows that the proposed method is effective for both low-dimensional and high-dimensional data. The performance of MKL-DR is worst among all algorithms, which validates that the SDP relaxation technique applied in MKL-DR has a negative influence on the performance of dimensionality reduction. The performance of MKL-TR is similar to that of MKL-SRTR, since MKL-SRTR only utilizes spectral regression to improve the speed of MKL-TR.

**Figure 1:**
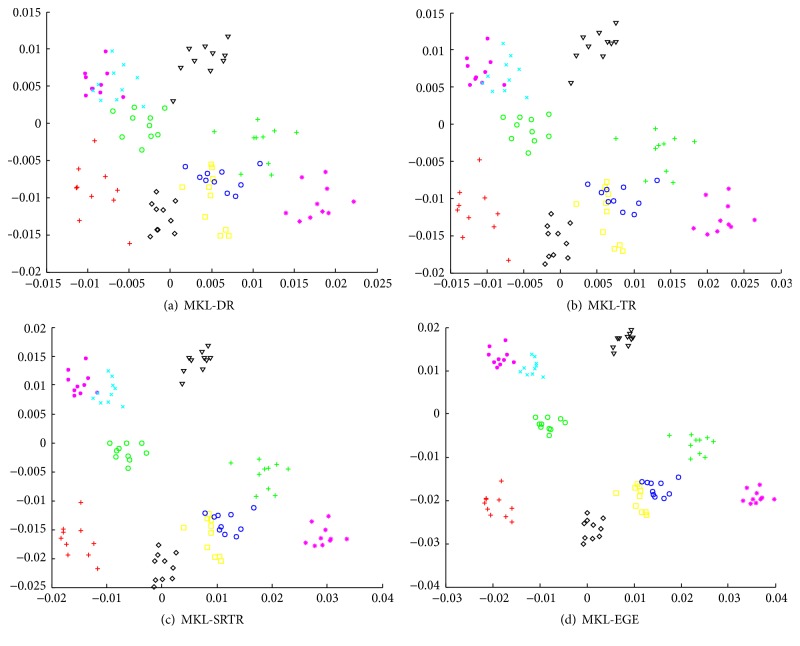
The two-dimensional visualizations of the embedding data from the first 10 classes of ORL. (a) The embedding data in the MKL-DR subspace; (b) the embedding data in the MKL-TR subspace; (c) the embedding data in the MKL-SRTR subspace; (d) the embedding data in the MKL-EGE subspace.

**Figure 2:**
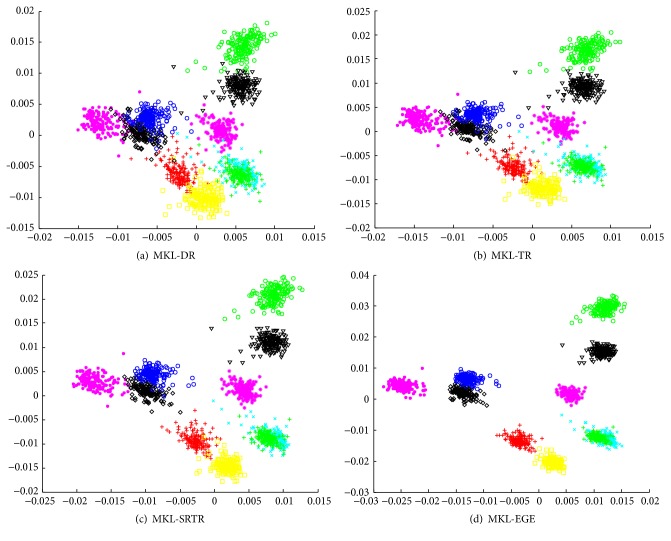
The two-dimensional visualizations of the embedding data from the first 10 classes of PIE. (a) The embedding data in the MKL-DR subspace; (b) the embedding data in the MKL-TR subspace; (c) the embedding data in the MKL-SRTR subspace; (d) the embedding data in the MKL-EGE subspace.

We used all samples from each class of ORL as training data and used different algorithms to obtain corresponding two-dimensional embedding results. To further validate and compare the final results among different algorithms, we also tested them on PIE, which has the maximum number of samples. The final embedding results are shown in Figures 1 and 2, respectively. As can be seen from Figures 1 and 2, the embedding data obtained by MKL-DR, MKL-TR, and MKL-SRTR is overlapped more seriously than MKL-EGE. The embedding data obtained by MKL-EGE has the best separability, which demonstrates that MKL-EGE is more effective than other algorithms for high-dimensional face data. Consequently, the performance of classification using SVM based on MKL-EGE is best compared to other algorithms.

**Figure 3:**
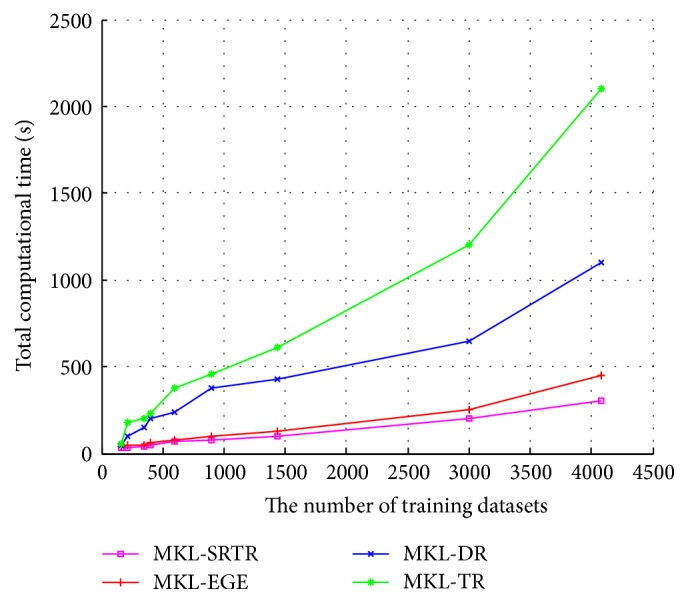
Time cost of different methods on all datasets versus different number of data examples.

**Figure 4:**
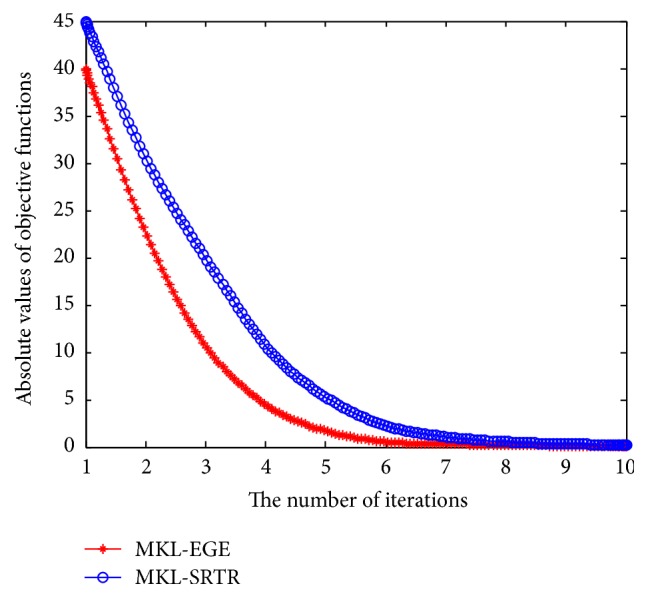
The convergence curves of MKL-EGE and MKL-SRTR.

**Figure 5:**
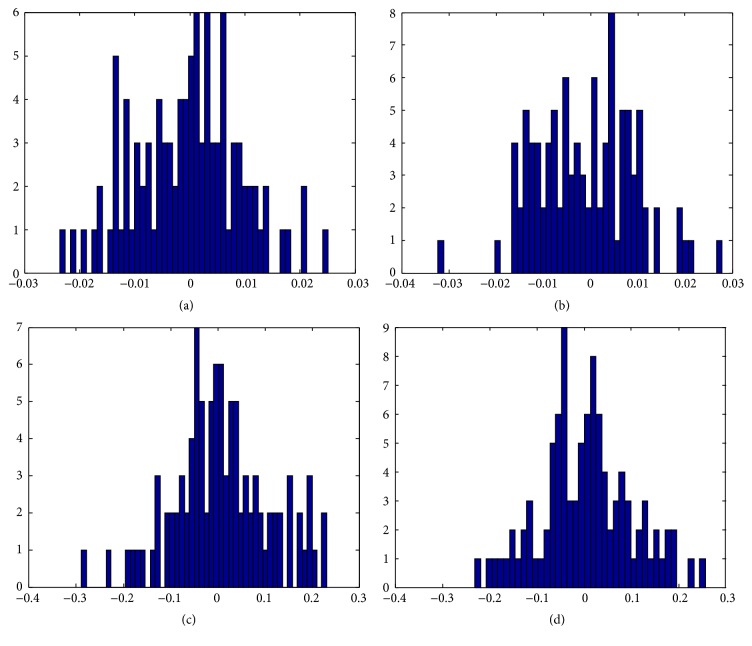
Histograms of the values of *f*(*r*) found by different algorithms on USPS. (a) MKL-DR; (b) MKL-TR; (c) MKL-SRTR; (d) MKL-EGE.

To compare the computational time of different algorithms, we used all data samples of each dataset as training data to perform different multiple kernel dimensionality reduction methods. The results are displayed in Figure 3. From Figure 3, we can see that MKL-SRTR and MKL-EGE are much faster than MKL-DR and MKL-TR. Since MKL-EGE utilizes a binary search in inner iterations to speed up convergence, its speed is only a little slower than that of MKL-SRTR for the sake of eigenvalue decomposition of dense matrices. The convergence curves of MKL-EGE and MKL-SRTR are displayed in Figure 4. As can be seen from Figure 4, the speed of convergence for MKL-EGE is faster than that of MKL-SRTR; this is due to the fact that MKL-SRTR needs to predefine step length of parameter *r* and does not adjust adaptively step length in each iteration. For comparing the approximation performances of different algorithms, Figure 5 shows the histograms of the *f*(*r*) values obtained by all algorithms in 100 runs. As can be seen from Figure 5, compared with other algorithms, the approximate solutions of MKL-EGE are more concentrated near zero, which validates that our algorithm can more effectively find the root *r* ^*∗*^ approximately. Overall, the proposed method is the most cost-efficient among all algorithms.

### 4.2. Experiments on Unsupervised Learning

To evaluate the performance of MKL-EGE in unsupervised settings, we first used all algorithms to project the original data onto a subspace, where the normalized cut spectral clustering (NC) [30] algorithm was performed to evaluate the clustering performance. For MKL-TR, we set **M** = **W** and **N** = diag(**W**1), where **W** is the affinity matrix for MKL-EGE, MKL-SRTR, and MKL-DR. In the unsupervised case, we set the number of clusters as the number of classes *c* in each dataset. In order to evaluate the clustering performance, the normalized mutual information (**NMI**) and Rand index (**RI**) [31] were adopted.

We used the same datasets and the same preprocessing procedure as in supervised learning experiments. For unsupervised MKL-DR, initializing **A** first obtained more stable performances. Thus, this strategy was adopted in the experiments. To obtain stable results, for each dataset, we computed the average results of each algorithm over 20 runs.

The values of** NMI** and** RI** obtained by these algorithms are reported in Tables 3 and 4, respectively. From Tables 3 and 4, we can see that MKL-EGE performs better than other algorithms in most datasets, which demonstrates that it can improve the performance of dimensionality reduction by using EGE and regularization terms. Consequently, it has the ability to find a more effective combination of base kernels in unsupervised settings. MKL-TR and MKL-SRTR evidently outperform MKL-DR, which indicates that the SDP relaxation used in MKL-DR also has a negative effect on the performance of dimensionality reduction in this case.

### 4.3. Experiments on Semisupervised Learning

In the semisupervised case, MKL-DR, MKL-TR, MKL-SRTR, and MKL-EGE are actually the multiple kernel extensions of the semisupervised discriminant analysis (SDA) [32–34]. Given *l* labeled data {(**x**_*i*_, *y*_*i*_)}_*i*=1_^*l*^ and *u* unlabeled data {**x**_*j*_}_*j*=*l*+1_^*l*+*u*^, SDA can be specified by two affinity matrices **W** = [*w*_*ij*_] and **W**′ = [*w*_*ij*_′], defined as follows [34]:

(25) wij=1nyi+δ·sij,if  yi=yj,δ·sij,otherwise,wij′=1l,if  both  xi  and  xj  are  labeled,0,otherwise,

where

(26) $$\begin{array}{c} s_{ij}=\left\{\begin{array}{cc} 1, & \text{if }i\in N_{k}\left(j\right)\text{ or }j\in N_{k}\left(i\right)\\ 0, & \text{otherwise} \end{array}\right. \end{array}$$

and 0 < *δ* ≤ 1 is the parameter to adjust the weight between the label information and unsupervised neighbor information. For MKL-TR, we set **M** = **D** − **W** and **N** = **D**′ − W′. *δ* is set as 0.1 for all algorithms.

In semisupervised settings, the same datasets and parameter initialization were used. We randomly selected one-half training data as labeled data for each dataset. Each algorithm was independently performed over 20 times. The average classification accuracies as well as the standard deviations are reported in Table 5. As can be seen from Table 5, the proposed MKL-EGE algorithm performs better than MKL-SRTR, MKL-TR, and MKL-DR. Our proposed algorithm, which effectively takes advantage of EGE and regularized trace ratio optimization, can automatically learn weights of base kernels and combine them to improve the performance of dimensionality reduction. By virtue of the same prior information, the proposed algorithm achieves 10 best results among 13 datasets compared with these state-of-the-art methods.

**Figure 6:**
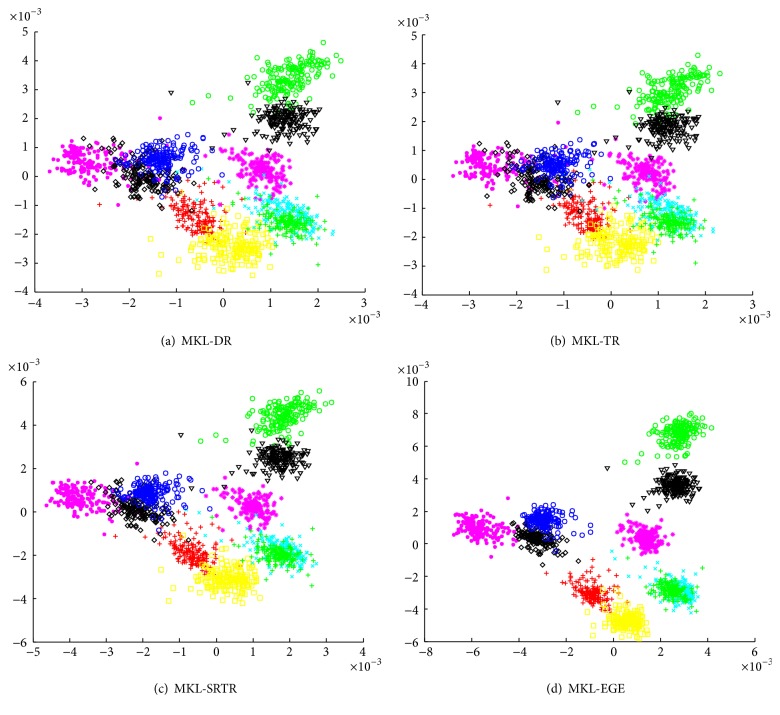
The two-dimensional visualizations of the embedding data from the first 10 classes of PIE. (a) The embedding data obtained by semisupervised MKL-DR; (b) the embedding data obtained by semisupervised MKL-TR; (c) the embedding data obtained by semisupervised MKL-SRTR; (d) the embedding data obtained by semisupervised MKL-EGE.

To visualize the semisupervised dimensionality reduction results, we used all samples from the first 10 classes of PIE and projected them into a two-dimensional subspace to generate a graphical representation, shown in Figure 6. From Figure 6, we can observe that the embedding data obtained by MKL-EGE and MKL-SRTR is separated from each other more clearly than MKL-DR and MKL-TR. The embedding data obtained by MKL-EGE has the best separability, which further validates that the performance of MKL-EGE is much better than that of other algorithms in the semisupervised case.

### 4.4. Experiments on Real World Datasets

To evaluate the effectiveness of MKL-EGE on real world datasets, it serves as a feature extraction method for bearing vibration signals, which were provided by bearing accelerometer sensors under different operating loads and bearing conditions from mines. The vibration signals were collected by using a 16-channel digital audio tape (DAT) recorder at the sampling frequency 12 kHz. Similar to the experimental settings in [35], the experimental vibration data were divided into four datasets, named as D_IRF, D_ORF, D_BF, and D_MIX shown in Table 6, where “07,” “14,” “21,” and “28” mean that fault diameter is 0.007, 0.014, 0.021, and 0.028 inches. We used one-half vibration data as training samples and another one-half as testing samples.

Similar to the experimental settings in [35], we firstly transformed the obtained vibration signals into 10 time domain features, 3 frequency domain features, and 16 time-frequency domain features. Secondly, low-dimensional features were extracted for performing bearings fault diagnosis or prognosis. Finally, SVM was used to evaluate the performance of different DR methods. The first three extracted features corresponding to the largest eigenvalues are employed as the input features of SVM. The classification accuracy rates are reported in Table 7. It can be observed that MKL-EGE achieves much better results compared to other algorithms on all datasets, which further demonstrates the effectiveness of our method for feature extraction of vibration signals in real applications.

## 5. Conclusion

In this paper, we propose a new multiple kernel dimensionality reduction method called MKL-EGE. By means of EGE and regularized trace ratio maximization, the proposed method not only avoids the SDP relaxation of MKL-DR but improves the performance of multiple kernel dimensionality reduction further. Moreover, the proposed algorithm makes good use of the binary search and alternative optimization scheme to efficiently find optimal solutions. Experimental results validate the effectiveness of this method. In the future, we plan to incorporate pair constraints into our framework and exploit multiple kernel dimensionality reduction via convex optimization.

## Figures and Tables

**Algorithm 1:**
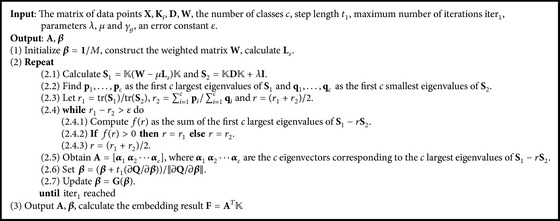
The proposed MKL-EGE algorithm.

**Table 1:** Description of experimental datasets.

Datasets	Dimensions	# of samples	# of classes
Ionosphere	33	351	2
Sonar	60	208	2
USPS	256	3000	10
Isolet	617	900	3
MINIST	784	600	3
Yale	1024	165	15
PIE	1024	4080	68
ORL	1024	400	40
COIL-20	1024	1440	20
20NG (comp)	28299	4852	5
20NG (rec)	24990	3968	4
20NG (sci)	30383	3945	4
20NG (talk)	29426	3250	4

**Table 2:** Classification accuracy of different DR methods.

Datasets	MKL-DR	MKL-TR	MKL-SRTR	MKL-EGE
Ionosphere	91.07 ± 1.69	**95.17 ± 0.89**	94.50 ± 3.67	94.64 ± 0.97
Sonar	83.90 ± 4.56	86.68 ± 3.47	**88.75 ± 2.83**	87.35 ± 5.46
USPS	93.45 ± 0.62	93.63 ± 0.53	92.73 ± 0.8	**96.43 ± 0.69**
Isolet	94.72 ± 0.25	96.50 ± 0.19	96.80 ± 0.11	**97.82 ± 0.19**
MINIST	91.29 ± 0.99	92.13 ± 0.92	92.64 ± 0.81	**96.13 ± 0.74**
Yale	76.54 ± 5.98	79.83 ± 4.19	78.83 ± 4.31	**82.83 ± 4.72**
PIE	92.41 ± 0.65	94.96 ± 0.11	94.83 ± 0.25	**97.97 ± 0.02**
ORL	90.94 ± 2.20	95.81 ± 1.02	94.63 ± 1.38	**96.32 ± 0.94**
COIL-20	91.87 ± 0.69	93.62 ± 0.35	92.55 ± 0.69	**95.70 ± 0.29**
20NG (comp)	86.00 ± 0.86	84.58 ± 1.05	85.48 ± 0.65	**89.73 ± 0.79**
20NG (rec)	94.02 ± 7.28	96.01 ± 0.66	95.87 ± 2.61	**97.85 ± 0.53**
20NG (sci)	95.19 ± 0.59	95.89 ± 0.60	96.36 ± 1.25	**98.21 ± 0.73**
20NG (talk)	91.22 ± 1.12	92.4 ± 0.98	93.24 ± 2.23	**95.78 ± 0.69**

**Table 3:** NMI of different dimensionality reduction algorithms for the clustering task.

Datasets	MKL-TR	MKL-SRTR	MKL-DR	MKL-EGE
Ionosphere	0.1336	0.1422	0.1292	**0.1829**
Sonar	0.0045	0.0004	0.0004	**0.0047**
USPS	0.5725	**0.6069**	0.5451	0.5771
Isolet	0.9474	0.9332	0.9237	**0.9612**
MINIST	0.7287	0.7342	0.7083	**0.7957**
Yale	0.5414	0.5989	0.5212	**0.6305**
PIE	0.0390	0.0629	0.0351	**0.1426**
ORL	0.7813	0.7753	0.7588	**0.8061**
COIL-20	**0.7095**	0.6995	0.6859	0.7034
20NG (comp)	0.3224	0.3173	0.3094	**0.5536**
20NG (rec)	0.7797	0.7497	0.7241	**0.8502**
20NG (sci)	0.7468	0.7372	0.6205	**0.8147**
20NG (talk)	0.3954	0.3489	0.3328	**0.5829**

**Table 4:** RI of different dimensionality reduction algorithms for the clustering task.

Datasets	MKL-TR	MKL-SRTR	MKL-DR	MKL-EGE
Ionosphere	0.5740	0.5762	0.5853	**0.6034**
Sonar	**0.5023**	0.5000	0.5000	0.5019
USPS	0.8690	**0.8869**	0.8619	0.8720
Isolet	0.9454	0.9497	0.9340	**0.9668**
MINIST	0.8822	0.8871	0.8870	**0.9087**
Yale	0.8942	0.9028	0.9009	**0.9215**
PIE	0.6338	0.6492	0.6335	**0.6953**
ORL	0.9536	0.9505	0.9517	**0.9798**
COIL-20	0.8945	0.8929	0.8945	**0.9075**
20NG (comp)	0.6883	0.6646	0.6648	**0.7897**
20NG (rec)	0.9249	0.9183	0.9172	**0.9662**
20NG (sci)	0.9072	0.9193	0.8157	**0.9316**
20NG (talk)	0.5655	0.6636	0.6210	**0.7239**

**Table 5:** Classification accuracies of different semisupervised DR methods.

Datasets	MKL-TR	MKL-DR	MKL-SRTR	MKL-EGE
Ionosphere	**82.31 ± 2.82**	80.36 ± 4.33	80.25 ± 3.24	81.02 ± 2.24
Sonar	**61.43 ± 4.45**	55.65 ± 4.87	60.75 ± 4.47	60.83 ± 3.16
USPS	83.58 ± 1.73	81.97 ± 1.25	82.74 ± 0.78	**86.13 ± 0.97**
Isolet	**94.26 ± 0.83**	91.44 ± 0.58	92.54 ± 0.65	93.05 ± 0.71
MNIST	89.68 ± 1.46	88.67 ± 1.43	90.46 ± 1.31	**92.32 ± 1.24**
Yale	35.43 ± 5.32	30.28 ± 4.38	32.29 ± 5.17	**47.08 ± 4.15**
PIE	62.69 ± 6.87	63.31 ± 5.52	64.13 ± 7.84	**68.67 ± 5.65**
ORL	50.13 ± 3.21	46.18 ± 5.19	51.25 ± 2.56	**59.26 ± 3.34**
COIL-20	71.92 ± 2.17	69.47 ± 2.34	70.31 ± 3.29	**75.95 ± 2.56**
20NG (comp)	75.34 ± 0.62	52.92 ± 5.30	76.74 ± 1.25	**82.19 ± 0.76**
20NG (rec)	92.66 ± 0.68	92.23 ± 0.40	93.37 ± 0.73	**94.46 ± 0.89**
20NG (sci)	91.39 ± 0.74	91.88 ± 0.42	91.95 ± 0.26	**93.94 ± 0.54**
20NG (talk)	82.48 ± 0.87	77.63 ± 14.21	84.06 ± 1.59	**88.62 ± 0.69**

**Table 6:** The experimental datasets.

Datasets	Number	Fault type and diameter	Description
D_IRF	1000	Normal, IRF07, IRF14, IRF21, IRF28	Inner race fault severity
D_ORF	800	Normal, ORF07, ORF14, ORF21	Outer race fault severity
D_BF	1000	Normal, BF07, BF14, BF21, BF28	Ball fault severity
D_MIX	800	Normal, IRF14, ORF14, BF14	Mixed fault classification

**Table 7:** The classification accuracy rates on four bearing vibration signal datasets.

Datasets	MKL-TR	MKL-DR	MKL-SRTR	MKL-EGE
D_MIX	0.9363	0.9257	0.9485	**0.9835**
D_IRF	0.9415	0.9151	0.9312	**0.9785**
D_ORF	0.9228	0.9238	0.9554	**0.9769**
D_BF	0.9086	0.8992	0.9027	**0.9394**

## Notations

*R*^*d*^*⁡*: The input *d*-dimensional Euclidean space
*n*⁡*⁡*: The number of total data points
*c*⁡*⁡*: The number of classes that the samples belong to
**X**⁡*⁡*: **X** = [**x**_1_,…, **x**_*n*_] ∈ *ℝ* ^*d*×*n*^ is the training data matrix
**Y**⁡*⁡*: **Y** = (**y**_1_,…, **y**_*n*_) ∈ *ℝ* ^*m*×*n*^ is the 0-1 label vector *y*_*i*_ is the lable of **x**_*i*_
*k*(**x**, **y**)*⁡*: Kernel function of data vectors **x** and **y**
**K**⁡*⁡*: Kernel matrix **K** = {*k*(**x**_*i*_, **x**_*j*_)} ∈ *ℝ* ^*n*×*n*^
{**K**_*m*_}_*m*=1_^*M*^*⁡*: Base kernels
**β***⁡*: **β** = [*β*_1_,…,*β*_*M*_]^*T*^ ∈ *ℝ* ^*M*^, representing nonnegative coefficients of base kernels
*𝕂*⁡*⁡*: The ensemble kernel *𝕂* = ∑_*m*=1_^*M*^ *β*_*m*_ **K**_*m*_
tr⁡(**M**)*⁡*: The trace of the matrix **M**, that is, the sum of the diagonal elements of the matrix **M**.

## References

[1] Li L., Goh W., Lim J. H., Pan S. J. (2014). Extended Spectral Regression for efficient scene recognition. *Pattern Recognition*.

[2] Nazarpour A., Adibi P. (2015). Two-stage multiple kernel learning for supervised dimensionality reduction. *Pattern Recognition*.

[3] Cai H., Mikolajczyk K., Matas J. (2011). Learning linear discriminant projections for dimensionality reduction of image descriptors. *IEEE Transactions on Pattern Analysis & Machine Intelligence*.

[4] Huang X., Lei Z., Fan M., Wang X., Li S. Z. (2013). Regularized discriminative spectral regression method for heterogeneous face matching. *IEEE Transactions on Image Processing*.

[5] Zhu X., Huang Z., Yang Y., Tao Shen H., Xu C., Luo J. (2013). Self-taught dimensionality reduction on the high-dimensional small-sized data. *Pattern Recognition*.

[6] Zhu X., Huang Z., Tao Shen H., Cheng J., Xu C. (2012). Dimensionality reduction by Mixed Kernel Canonical Correlation Analysis. *Pattern Recognition*.

[7] Jolliffe I. T. (1986). *Principal Component Analysis*.

[8] Cai D., He X., Han J. Semi-supervised discriminant analysis.

[9] Tenenbaum J. B., de Silva V., Langford J. C. (2000). A global geometric framework for nonlinear dimensionality reduction. *Science*.

[10] Roweis S. T., Saul L. K. (2000). Nonlinear dimensionality reduction by locally linear embedding. *Science*.

[11] Belkin M., Niyogi P. (2001). Laplacian eigenmaps and spectral techniques for embedding and clustering. *Advances in Neural Information Processing Systems*.

[12] He X., Niyogi P. Locality preserving projections.

[13] Yan S., Xu D., Zhang B., Zhang H.-J., Yang Q., Lin S. (2007). Graph embedding and extensions: a general framework for dimensionality reduction. *IEEE Transactions on Pattern Analysis and Machine Intelligence*.

[14] Bengio Y., Paiement J.-F., Vincent P., Delalleau O., Roux N. L., Ouimet M. (2003). Out-of-sample extensions for LLE, isomap, MDS, eigenmaps, and spectral clustering. *Advances in Neural Information Processing Systems 16*.

[15] Ham J., Lee D. D., Mika S., Schölkopf B. A kernel view of the dimensionality reduction of manifolds.

[16] De Silva V., Tenenbaum J. B. (2003). Global versus local methods in nonlinear dimensionality reduction. *Advances in Neural Information Processing Systems 15 (NIPS '02)*.

[17] Brand M. Continuous nonlinear dimensionality reduction by kernel eigenmaps.

[18] Liu B., Xia S.-X., Meng F.-R., Zhou Y. (2015). Extreme spectral regression for efficient regularized subspace learning. *Neurocomputing*.

[19] Cortes C., Mohri M., Rostamizadeh A., Bengio Y., Schuurmans D., Lafferty J., Williams C. K. I., Culotta A. (2009). Learning non-linear combinations of kernels. *Advances in Neural Information Processing Systems*.

[20] Lin Y.-Y., Liu T.-L., Fuh C.-S. (2011). Multiple kernel learning for dimensionality reduction. *IEEE Transactions on Pattern Analysis and Machine Intelligence*.

[21] Jiang W., Chung F.-L. (2014). A trace ratio maximization approach to multiple kernel-based dimensionality reduction. *Neural Networks*.

[22] Liu M., Sun W., Liu B. (2015). Multiple kernel dimensionality reduction via spectral regression and trace ratio maximization. *Knowledge-Based Systems*.

[23] Cai D., He X., Han J. Spectral regression for efficient regularized subspace learning.

[24] Cai D., He X., Zhang W. V., Han J. Regularized locality preserving indexing via spectral regression.

[25] Belkin M., Niyogi P., Sindhwani V. (2006). Manifold regularization: a geometric framework for learning from labeled and unlabeled examples. *Journal of Machine Learning Research*.

[26] Nie F., Zeng Z., Tsang I. W., Xu D., Zhang C. (2011). Spectral embedded clustering: a framework for in-sample and out-of-sample spectral clustering. *IEEE Transactions on Neural Networks*.

[27] Ngo T. T., Bellalij M., Saad Y. (2012). The trace ratio optimization problem. *SIAM Review*.

[28] Wang H., Yan S., Xu D., Tang X., Huang T. Trace ratio vs. ratio trace for dimensionality reduction.

[29] Chang C.-C., Lin C.-J. (2011). LIBSVM: a library for support vector machines. *ACM Transactions on Intelligent Systems and Technology*.

[30] Xiang S., Nie F., Zhang C. (2008). Learning a Mahalanobis distance metric for data clustering and classification. *Pattern Recognition*.

[31] Chen W., Feng G. (2012). Spectral clustering: a semi-supervised approach. *Neurocomputing*.

[32] Chen H.-T., Chang H.-W., Liu T.-L. Local discriminant embedding and its variants.

[33] Cai D., He X., Han J. Efficient Kernel Discriminant Analysis via spectral regression.

[34] Cai D., He X., Han J. (2008). SRDA: an efficient algorithm for large scale discriminant analysis. *IEEE Transactions on Knowledge and Data Engineering*.

[35] Xia Z., Xia S., Wan L., Cai S. (2012). Spectral regression based fault feature extraction for bearing accelerometer sensor signals. *Sensors*.

